# Innovative and sustainable solutions for reducing ‘zero-dose’ vaccination: How can the region respond?

**DOI:** 10.3389/fpubh.2025.1652977

**Published:** 2025-09-16

**Authors:** Zeina Abdel Majeed, Nada Ahmad, Osama Mere, Saadia Farrukh, Ezzeddine Mohsni, Mohamed Derow, Adriana Cuen, Mona Mohamed, Mohamedanas Patni, Magid Al Gunaid, Yousef Khader, Mohannad Al Nsour, Haitham Bashier

**Affiliations:** ^1^EMPHNET, Amman, Jordan; ^2^WHO EMRO, Nasr City, Egypt; ^3^UNICEF MENARO, New York, NY, United States; ^4^Ministry of Health Somalia, Somalia, Somalia; ^5^GAVI, Geneva, Switzerland; ^6^RAK Medical & Health Sciences University, Ras Al-Khaimah, United Arab Emirates; ^7^Department of Public Health, Jordan University of Science and Technology, Irbid, Jordan

**Keywords:** zero dose, immunization, vaccination, sustainable, innovative

## Abstract

**Background:**

This roundtable focused on the challenge of reaching zero-dose children in the Eastern Mediterranean Region (EMR) amid declining immunization rates and increasing health disparities. ‘Zero-dose’ children are concentrated in high-conflict or underserved areas, such as Pakistan, Afghanistan, Sudan, and Yemen, making them particularly vulnerable to vaccine-preventable diseases. This discussion convened key stakeholders, including public health experts, EPI managers, and representatives from organizations like WHO, Gavi, and CDC, to explore effective, sustainable solutions for reducing ‘zero-dose’ prevalence in the EMR.

**Purpose:**

The roundtable aimed to assess regional barriers to immunization, examine country-specific initiatives to reach ‘zero-dose’ children, and recommend targeted strategies that align with global immunization goals, such as the Immunization Agenda 2030.

**Method:**

Through expert presentations, panel discussions, and participant interaction, attendees analyzed key causes of ‘zero-dose’ prevalence, focusing on conflict-related disruptions, health system fragility, and socioeconomic challenges. Comparative insights were drawn from other regions, highlighting adaptable solutions from Sub-Saharan Africa and other high-burden areas.

**Results:**

Findings indicated that conflicts, infrastructure limitations, and social barriers are major drivers of low vaccination rates. Approximately 2.85 million ‘zero-dose’ children were identified in the EMR, primarily concentrated in a few high-burden countries. Country-specific efforts, such as the “Big Catch-Up” campaign, were acknowledged for their impact, but gaps in sustainable funding and operational capacity remain. Strategies focusing on community engagement, data-driven microplanning, and integration of immunization with broader health services were recommended to overcome access challenges.

**Conclusion:**

To reach ‘zero-dose’ children, coordinated, tailored approaches are essential. Community-driven microplanning and enhanced political and financial support can bolster immunization efforts in fragile settings. The roundtable underscored the role of primary health systems in addressing vaccination gaps, contributing to universal health coverage, and fostering resilience in conflict-affected areas. Future policies should prioritize collaboration among sectors, sustainable funding, and innovative outreach to achieve immunization equity across the EMR.

## Introduction

### Defining ‘zero-dose’ children and their significance

The term ‘zero-dose children’ is used to describe children who have not received a single dose of a specific vaccine of interest or to describe children who have not received any vaccine at all. The percentage of ‘zero-dose’ children for diphtheria, tetanus, and pertussis-containing (DTP) vaccines is commonly used as a proxy for lack of access to routine immunization services. According to WHO/UNICEF estimates, more than 14 million children worldwide were ‘zero-dose’ for DTP in 2022, with 9.1% of these cases occurring in the Eastern Mediterranean Region (EMR). This is a decrease compared to the 18.1 million children globally in 2021 (1). In 2022, about 20.5 million infants did not receive essential vaccines (2). This marks a decrease from the peak of 25 million children reported as ‘zero-dose’ for DTP in 2021, the highest number since 2009. Additionally, there was an increase of 5 million completely unvaccinated children in 2021 compared to the onset of the COVID-19 pandemic in 2019 (1).

‘Zero-dose’ children face heightened susceptibility to vaccine-preventable diseases, leaving them with little or no protection against severe illness, disability, or death caused by these infections. Beyond individual risk, these children also pose a significant public health concern, as their vulnerability increases the likelihood of disease transmission within communities, particularly to other unvaccinated or under-immunized children. This dual risk underscores the importance of identifying and reaching ‘zero-dose’ populations to strengthen routine immunization coverage and interrupt preventable disease outbreaks.

#### Regional burden: immunization coverage in the EMR

In 2023, there were approximately 2.85 million ‘zero-dose’ children in the EMR, a significant increase from the previous year’s estimate. This rise reflects a broader regional decline in immunization coverage due to factors including socioeconomic challenges, political instability, and disruptions caused by the COVID-19 pandemic. Specifically, DTP1 coverage in the EMR fell to 85%, and DTP3 coverage dropped to 79%, leaving nearly 4 million children vulnerable to vaccine-preventable diseases (3). An estimated 80% of these ‘zero-dose’ children live in Gavi-eligible countries, including Afghanistan, Pakistan, Somalia, Sudan, and Yemen (4). These countries encompass a diversity of settings with anticipated variability in the drivers of ‘zero-dose’ and under-immunized children, which are complex, interrelated, and highly contextualized (5) ‘Zero-dose’ children in Pakistan alone constitute around 14% of the total ‘zero-dose’ in EMR (3).

### Challenges to immunization in high-burden countries

The top 10 EMR countries, including Pakistan, Afghanistan, Sudan, and Yemen, account for 98% of the region’s ‘zero-dose’ children. Moreover, several EMR countries still fall short of the WHO-recommended 90% DTP1 vaccination target, primarily due to systemic challenges, including weak health systems, conflict, and geographical barriers. These “missed communities” often coincide with higher rates of other health disparities, such as limited access to maternal, neonatal, and child health services (6). Several countries in the region did not reach the 90% DTP vaccination target due to multiple factors including suboptimal vaccination coverage in hard-to-reach subpopulations, non-utilization of antenatal care services, younger mothers, being the 3rd or higher born child in the family, lack of access to vaccination information, longer distances from vaccination facilities, and children living in pockets of communities that traditionally miss primary healthcare services, including immunization—the so-called missed communities (7). Communities with a high proportion of ‘zero-dose’ children tend to have girls who are not in school, high rates of violence against women, and lack of contraceptive, reproductive, maternal, neonatal, and pediatric health services (8). These missed communities are often the epicenters of disease outbreaks (e.g., polio, yellow fever, measles, meningitis, cholera, Ebola virus) and can thus be valuable targets for prevention efforts (9). The most deprived group represents children who were not born in a health facility, to a mother who did not receive antenatal care visits and accordingly did not receive tetanus vaccine before or during pregnancy (2).

Because women and mothers are primary caregivers for children and play a key role in their children’s immunization, empowering women gives them the autonomy and ability to make informed decisions, including seeking care and health services for their children (10). Making progress toward health equity requires a granular understanding of where there are gaps in access or uptake, why those gaps exist, and what can be done to address them, especially since 50% of ‘zero-dose’ children live in three critical geographic contexts: urban areas, remote communities, and populations in conflict settings where they do not only lack access to vaccines but lack access to other essential child health services (4).

### Policy frameworks and strategic responses

Immunization Agenda 2030 sets a global vision and strategy for vaccines and immunization, committing to an ambitious target of reducing the number of ‘zero-dose’ children by 50% by 2030 (11). As the world’s most widely available health intervention, childhood immunization can be leveraged to strengthen primary health care for missed communities, bringing us closer to universal health coverage (UHC) (11) Therefore, reaching ‘zero-dose’ children will require tailored approaches that address multiple and intersecting sociocultural barriers, economic vulnerabilities, and health system challenges to deliver immunization services through the primary health care system (12).

The COVID-19 pandemic has disrupted health services and preventive interventions, including childhood immunizations, and new efforts are critically needed to ensure no one is left behind (13). Reaching ‘zero-dose’ children and missed communities with health services like routine immunization is a crucial goal of UHC, the Immunization Agenda 2030, and the Gavi Alliance’s 2021–2025 Strategy (14) Focusing on ‘zero-dose’ children is particularly important because those reached with the first vaccine are highly likely to receive the remaining vaccines (14). Health for all means getting those left furthest behind and reaching them with life-saving vaccines could be the pathway to providing other health services (4). Countries’ roll-out of COVID-19 vaccines offered a historic opportunity to strengthen routine immunization, reach ‘zero-dose’ and missed communities with an entire course of vaccines, including with primary healthcare services, and build resilience to future emergencies (14).

### The roundtable’s role in addressing gaps

The Eastern Mediterranean Public Health Network (EMPHNET) held its Eighth Regional Conference in Amman, Jordan on the 15th through the 18th of September 2023 in Amman under the theme “Advancing Public Health Preparedness and Response: Challenges, Opportunities, and Ways Forward.” The conference sessions in the form of workshops, forums, and roundtables addressed challenges and identified opportunities to advance public health preparedness and response in the Eastern Mediterranean Region and beyond. The roundtable entitled “Innovative and Sustainable Solutions for Reducing ‘Zero-Dose’ Vaccination: How Can the Region Respond?” This roundtable aimed to assess the current regional status of ‘zero-dose’ children, explore country-specific efforts and initiatives to reach them, and facilitate the exchange of experiences, best practices, and lessons learned. Participants also sought to recommend actionable strategies for sustainable solutions to reduce the number of ‘zero-dose’ children, aligning with global and regional targets.

## Roundtable description

This roundtable holds critical significance in addressing the challenge of reaching ‘zero-dose’ children, particularly in the EMR. By bringing together stakeholders from various countries, this roundtable provided a platform for sharing best practices, experiences, and innovative approaches to effectively reach these hard-to-reach populations. Moreover, the roundtable served as an opportunity to align regional and national efforts with global initiatives such as the Immunization Agenda 2030, Gavi’s 2021–2025 Strategy, and the goals of universal health coverage (UHC). Given that the COVID-19 pandemic has disrupted health systems and immunization services worldwide, this roundtable was timely and essential for renewing commitments, identifying new strategies, and ensuring that no child is left behind. The discussions focused on sustainable solutions to reach ‘zero-dose’ children while aligning immunization efforts with the Immunization Agenda 2030, which envisions universal vaccination coverage by 2030.

The insights and recommendations generated from this roundtable are expected to influence policy and advocacy work across the region, leading to targeted actions that improve immunization equity and health outcomes. By prioritizing ‘zero-dose’ children, the roundtable also addressed broader public health concerns, including the prevention of disease outbreaks, the empowerment of women, and the strengthening of primary healthcare systems in underserved communities.

The roundtable considered several questions: Based on existing evidence, what are the leading underlying causes of ‘zero-dose’ children in the EMR, considering both supply and demand factors? Are there potential causes from similar contexts in other regions that require further study and analysis? Is there sufficient attention to ‘zero-dose’ children at the national and sub-national levels in the EMR regarding best practices that could define the challenge, lead advocacy efforts, and implement effective responses? What current country-based efforts and initiatives are underway to overcome the ‘zero-dose’ problem? What actions should be taken to ensure services are available when and where needed, including the involvement of different stakeholders, such as the private sector? How can we ensure vaccine supply accounts for the increase in missed vaccinations? What are the best strategies for engaging parents, caregivers, and communities, including private sector involvement? Who are the key players that can contribute to overcoming the ‘zero-dose’ challenge? How can we improve coordination among various sectors and stakeholders to effectively reach the unreached? What critical system elements beyond service delivery should be considered, and what potential improvements can be made? How can we balance innovation and sustainability in planning efforts to reach ‘zero-dose’ children?

## Methodology

Roundtable attendees included almost 100 participants of different public health professionals, Ministries of health leaders, EPI managers, EPI partners as well as FETP graduates and residents, researchers, and other health professionals from the region and beyond The official language of the Roundtable was English. The session started with an introduction to the topic and the panelists, followed by a 20-min presentation providing a general overview. The moderator then posed questions to the panelists organized into two rounds, focusing first on the causes of ‘zero-dose’ children and then on strategic solutions. Each round was followed by 20 min for participant questions, along with panel interactions, audience reflections, and clarifications, culminating in a wrap-up and conclusion.

Opening Presentation (20 min): A foundational overview of ‘zero-dose’ challenges in the EMR, including regional data and key barriers.

Panel Discussions: Structured into two thematic rounds:

- Root Causes: Analysis of supply- and demand-side drivers (e.g., conflict, gender disparities, health system gaps).
- Solutions: Strategies for equity, innovation (e.g., digital tools), and stakeholder coordination.

Audience Engagement: Two 20-min Q&A sessions allowed participants to share country-specific experiences and refine recommendations.

Synthesis: The moderator summarized actionable insights, aligning proposals with global frameworks like Immunization Agenda 2030 and Gavi’s 2021–2025 Strategy.

This participatory approach ensured evidence-based, context-specific recommendations to reduce ‘zero-dose’ disparities in the EMR.

## Findings

The roundtable took a comprehensive approach, blending expert presentations, panel discussions, and audience input to analyze immunization challenges and regional data on ‘zero-dose’ children in the Eastern Mediterranean Region (EMR). The first presentation provided a foundational overview, highlighting the growing number of children who have not received the first dose of DTP (DTP1) and the high concentration of ‘zero-dose’ children in priority countries. Data analysis revealed a troubling decline in DTP coverage across the EMR, with DTP1 coverage falling to 85% in 2023, leaving over 2.85 million children without this critical first dose. DTP3 coverage was similarly low, at 79%, meaning nearly 4 million children remain at risk for preventable diseases. The presentation identified major barriers contributing to these low coverage rates, including ongoing conflicts, health infrastructure limitations, and large populations displaced from areas with adequate healthcare access. Pakistan, with more than a million ‘zero-dose’ children, emerged as the largest contributor, followed by Sudan and Afghanistan, where both conflict and logistical challenges further hinder vaccination efforts.

Further discussions highlighted key causes of the ‘zero-dose’ issue in the EMR, focusing on how conflict, fragile health systems, and healthcare inaccessibility, particularly in remote areas, drive ‘zero-dose’ rates. One of the panelists emphasized the impact of sociopolitical instability, which disrupts healthcare delivery and erodes community trust in health services. In nations like Sudan, Yemen, Somalia, and Palestine, conflicts and system weaknesses severely impact vaccination efforts. Displacement and crises make microplanning and funding support insufficient to meet these needs, and both social and logistical barriers, including poor access to healthcare and low vaccine demand, compound the challenge.

Comparative insights from similarly affected regions, such as Sub-Saharan Africa, were explored, showcasing the effectiveness of integrated health campaigns that address multiple health needs beyond vaccination alone. For Somalia, incorporating essential services like maternal and child health into immunization outreach, especially in crisis settings, as seen in polio eradication efforts was recommended. Lessons from these regions underscored the importance of coordinated services and adaptable strategies to overcome challenges in conflict and displacement contexts.

Country-specific efforts, including the “Big Catch-Up” campaigns in Somalia, Yemen, and Afghanistan, aim to bridge immunization gaps. However, one speaker from the CDC stressed that these initiatives need robust political commitment and sustained financial resources to meet high operational costs. ‘Zero-dose’ children, particularly at sub-national levels, require more targeted attention and prioritization, which calls for stronger advocacy, political commitment, and resource allocation. Efforts like “The Big Catch-Up” and integrated outreach campaigns are already underway, but there is a pressing need to enhance data utilization for effective microplanning and coverage improvement.

The roundtable emphasized community engagement as essential for service availability, advocating for a bottom-up approach that empowers community leaders and local health teams. Participants discussed the potential benefits of private sector involvement and integrating vaccination with other health services to strengthen outreach, particularly in underserved regions. Ensuring adequate vaccine supplies, especially in areas with high numbers of missed children, depends on robust data systems to identify gaps and ensure sufficient vaccine stocks in conflict-affected regions. Sustaining these supply chains requires consistent political and financial commitment.

Strategies to engage parents, caregivers, and communities were also discussed, with a focus on localized outreach involving community leaders, academia, and civil society organizations. Integrated health campaigns supported by private sector engagement were suggested to increase acceptance and participation. Key stakeholders identified as instrumental to overcoming the ‘zero-dose’ challenge include community leaders, local health teams, academia, civil society, and organizations like Gavi. Effective collaboration among sectors and stakeholders is critical to reach those who remain unreached.

In addition to service delivery, the roundtable highlighted the need to strengthen data systems, improve community engagement, and integrate broader health services. Building resilient health systems that can withstand conflict and humanitarian crises is essential. The discussions also emphasized innovation and sustainability, calling for investments in data-driven approaches to identify ‘zero-dose’ children and flexible strategies to meet diverse needs. Long-term financial and political commitment is vital to support immunization efforts in complex settings. One of the panelists from WHO underscored the role of grassroots engagement, noting that involving local leaders and health workers in vaccination outreach could boost community acceptance and participation. Engaging the private sector was also seen as crucial for expanding service reach, especially in underserved areas.

To summarize these findings into short-term and long-term solutions, policy-based and operational strategies.

## Short-term solutions

### Operational strategies

Integrated health campaigns: Combining vaccination with other essential services (e.g., maternal and child health) in crisis settings (e.g., Somalia’s polio eradication model).

“Big Catch-Up” initiatives: Immediate immunization drives in high-burden countries (e.g., Somalia, Yemen, Afghanistan).

Localized outreach: Engaging community leaders, civil society, and academia to boost vaccine acceptance.

Private sector involvement: Leveraging private providers to expand service delivery in underserved areas.

Microplanning improvements: Using existing data to target ‘zero-dose’ children at sub-national levels.

### Policy-based strategies

Advocacy for urgent funding: Securing short-term financial support for high-operational-cost campaigns.

Emergency vaccine supply chains: Ensuring consistent stock availability in conflict zones.

## Long-term solutions

### Operational strategies

Strengthening data systems: Improving real-time tracking of ‘zero-dose’ children and coverage gaps.

Resilient health systems: Building infrastructure to withstand disruptions (e.g., conflict, displacement).

Integrated service delivery: Sustaining linkages between immunization and broader healthcare (e.g., nutrition, WASH).

Community empowerment: Long-term grassroots engagement to sustain trust and demand.

### Policy-based strategies

Political commitment: National policies prioritizing ‘zero-dose’ children with dedicated budgets.

Cross-sector collaboration: Formalizing partnerships with Gavi, WHO, civil society, and private sector.

Conflict-sensitive policies: Adapting immunization strategies for fragile states (e.g., Sudan, Yemen).

Sustainable financing: Establishing long-term funding mechanisms (e.g., domestic resource mobilization, donor alignment).

## Discussion

### Key discussion points

The challenges highlighted by experts as well as the roundtable participants across different regions underscore the complexity of immunization efforts in areas affected by conflict, poor infrastructure, and social barriers. These issues are not unique to one region but manifest differently across countries, necessitating context-specific approaches to address them effectively.

In Afghanistan, experts noted that low community awareness, poor communication, and security issues continue to hinder vaccination efforts. Despite improvements in security, there is a rise in ‘zero-dose’ cases, which suggests that physical access is not the only barrier. Sustainable programs are needed to support routine immunization, and better coordination of maternal and other health services could help integrate immunization into broader healthcare services (15). This aligns with the broader understanding that integrating maternal and child health services with immunization campaigns is critical in resource-constrained settings (16).

Sudan faces persistent supply chain and service delivery issues, particularly in sustaining large-scale vaccination campaigns like the Big Catch-Up. Logistical challenges in ensuring cold-chain storage and transportation in a country with ongoing political and economic instability complicate vaccine delivery (17) The challenges in Sudan reflect the need for strong logistical frameworks to support immunization in fragile states (15).

In Palestine, socioeconomic and cultural challenges limit access to vaccines. Participants emphasized that no single intervention could solve these problems, highlighting the importance of integrated approaches. This is supported by the broader literature, which underscores that healthcare services in conflict areas must be adaptable and inclusive of the socio-cultural context to effectively increase vaccine uptake (18).

Somalia presents another example where a “one-size-fits-all” approach does not work. Tailored strategies are essential to reach marginalized communities, especially nomadic populations (14). Community engagement and microplanning were consistently emphasized as critical in reaching the most vulnerable populations in such settings. Participants pointed out that national ownership and leadership are crucial for success, with communities being involved from the outset in microplanning to address the social drivers of low vaccine demand. Research highlights that community engagement is key to successful immunization campaigns, especially in fragile states where trust in healthcare systems is low (19).

Tailored approaches are critical to addressing the unique challenges faced by different regions. Participants emphasized the importance of subnational coverage, noting that accurate data is needed to identify and address coverage gaps. Accurate data collection is particularly important in regions with poor health infrastructure, where vaccination coverage may vary dramatically across districts or communities (6).

Participants’ observations regarding the increase in ‘zero-dose’ cases in Afghanistan, despite improved security, point to the need for deeper community-level investigations to understand why certain populations remain unreached. This is consistent with findings that ‘zero-dose’ children often reside in geographically isolated or politically marginalized communities (16). Without a detailed understanding of these social drivers, vaccination efforts may fail to reach their intended targets.

Leadership and national ownership are also critical for the success of immunization campaigns. Panelists emphasized that engaging communities from the outset in microplanning is essential for addressing the social factors that influence vaccine demand. This reflects the broader understanding that community participation in planning processes is essential for addressing cultural and social barriers to immunization (6, 15).

### Stakeholder perspectives

Several recommendations emerged from the discussion, reflecting the need for both innovative and sustainable solutions. Panelists from GAVI emphasized the importance of tailored, country-specific approaches, which could be supported through community engagement and technical assistance. This approach has been successfully implemented in Lebanon, where context-specific interventions have significantly improved vaccination coverage (15).

Panelists from CDC highlighted the importance of innovative approaches for conflict-affected areas, suggesting that microplanning should be informed by local insights. This recommendation is consistent with the need for flexible and adaptable approaches to immunization in fragile and conflict-affected settings, where logistical constraints and community trust issues may be significant barriers (18).

Panelists from WHO stressed the importance of community mapping and logistical planning to ensure that unvaccinated children are brought into the system. Detailed community mapping can provide essential insights into the social and cultural factors that contribute to low vaccination rates (19).

Panelists from GAVI emphasized the role of cost-effective digital solutions for data collection, alongside the need for stronger health systems to ensure the sustainability of vaccination efforts. Digital tools can provide real-time data that allow health workers to adapt their strategies and ensure that no community is left behind (16).

### Consensus and divergence

Finally, national ownership, government support, and the involvement of National Immunization Technical Advisory Groups (NITAGs) are critical for influencing decision-making and ensuring the long-term success of vaccination programs. As WHO panelists pointed out, engaging communities early in the planning process is essential to address the social drivers of vaccine uptake.

## Conclusion

The roundtable’s findings demand urgent, coordinated action to reach ‘zero-dose’ children in the Eastern Mediterranean Region, calling on policymakers and governments to strengthen subnational capacity through health worker training and service integration, secure sustainable financing by increasing domestic funding and aligned donor support, and deploy digital tools like GIS mapping for real-time tracking of underserved populations; international partners (Gavi, WHO, UNICEF, CDC) must scale integrated campaigns combining vaccination with nutrition/WASH services while providing context-specific technical assistance, mirroring successful models like Somalia’s polio eradication; local communities and civil society should lead microplanning efforts and grassroots advocacy to address cultural barriers, while the private sector and academia innovate portable cold-chain technologies and research on vaccine hesitancy drivers in conflict zones—all aligned with the Immunization Agenda 2030 target of halving ‘zero-dose’ children by 2030. These efforts will not only close immunization gaps but also strengthen primary healthcare systems, prevent outbreaks, and advance universal health coverage across the region, ensuring no child is left behind in the decade of action for vaccines.

## Future directions

Future directions must address critical gaps in sustaining community engagement during protracted crises and measuring the long-term impact of integrated service delivery, with research focusing on developing cost-effective delivery models for conflict-affected settings, creating scalable trust-building strategies (such as local influencer networks), and designing adaptive data systems that function despite infrastructure breakdowns - all of which will require collaborative research partnerships between governments, NGOs, and academic institutions to implement effectively.

## Data Availability

The original contributions presented in the study are included in the article/supplementary material, further inquiries can be directed to the corresponding authors.
